# Serum Soluble CD14 is Associated with Insulin Resistance and Cortical Inflammation in Nonhuman Primates Consuming Western versus Mediterranean Diets

**DOI:** 10.1002/alz.093270

**Published:** 2025-01-09

**Authors:** Brett M. Frye, Jacob D. Negrey, Courtney L. Sutphen, Suzanne Craft, Carol A. Shively, Thomas C. Register

**Affiliations:** ^1^ Wake Forest Alzheimer's Disease Research Center, Winston‐Salem, NC USA; ^2^ Wake Forest University School of Medicine, Winston‐Salem, NC USA; ^3^ Emory and Henry College, Emory, VA USA; ^4^ University of Arizona, Tucson, AZ USA

## Abstract

**Background:**

Diet composition is associated with neurodegenerative disease risk including Alzheimer’s Disease (AD). The adverse effects of Western‐style diets may be moderated, in part, by systemic as well as central inflammation, whereas the neuroprotective effects of Mediterranean diets may work through mechanisms that promote anti‐inflammatory phenotypes. Systemic inflammation also may induce insulin resistance, another risk factor for AD. However, controlled trials of human dietary patterns are not available nor has the impact of such diet patterns on systemic and central inflammation been examined in nonhuman primates.

**Method:**

Here we determine the effects of long term (31 months) consumption of a Mediterranean (n=16) or Western (n=21) diet in cynomolgus monkeys (Macaca fascicularis). We examined relationships between the circulating marker of monocyte activation soluble CD14 (sCD14) and lateral temporal cortical transcriptional profiles associated with proinflammatory (Cyclin dependent kinase 14 [CDK14]), and anti‐inflammatory/neuroprotective (lunatic fringe [LFNG], mannose receptor C type 2 [MRC2], solute carrier family 3 member 2 [SLCA32], butyrophilin subfamily 2 member A1 [BTN2A1], katanin regulatory subunit B1 [KATNB1], transmembrane protein 268 [TMEM268]) transcripts. We also analyzed associations between sCD14 and insulin and glucose responses in an intravenous glucose tolerance test (ivGTT).

**Result:**

On average, individuals in the Mediterranean diet group showed decreased signatures of monocyte activation, reflected by lower levels of circulating sCD14 compared to the Western group (mean difference±SE: 137.2±70.01, t=1.960, p=0.058). Serum sCD14 was negatively associated with anti‐inflammatory/neuroprotective brain transcripts: SL3CA2 (R=‐0.359, p=0.029), MRC2 (R=‐0.437, p=0.007), TMEM268 (R=‐0.304, p=0.067), BTN2A1 (R=‐0.300, p=0.071). sCD14 also was positively related to fasting insulin levels (R=0.401, p=0.021) and insulin area under the curve (R=0.350, p=0.046) in the ivGTT.

**Conclusion:**

These results demonstrate relationships between sCD14, a circulating marker of monocyte activation, insulin resistance, and a neuroinflammatory transcriptional profile in the temporal cortex. Mediterranean diet consumption was previously shown in these subjects to significantly alter circulating monocyte profiles toward an anti‐inflammatory phenotype and to preserve insulin sensitivity relative to Western diet consumption. The findings support multisystem anti‐inflammatory effects of Mediterranean diet consumption which may be mediated via integrated pathways. This work captures the complex interplay of diet composition, insulin resistance, inflammation, and brain health.

